# Risk assessment models to evaluate the necessity of prostate biopsies in North Chinese patients with 4-50 ng/mL PSA

**DOI:** 10.18632/oncotarget.14214

**Published:** 2016-12-26

**Authors:** Jing Zhao, Shuai Liu, Dexuan Gao, Sentai Ding, Zhihong Niu, Hui Zhang, Zhilong Huang, Juhui Qiu, Qing Li, Ning Li, Fang Xie, Jilei Cui, Jiaju Lu

**Affiliations:** ^1^ Department of Urology, Shandong Provincial Hospital Affiliated to Shandong University, Jinan, People's Republic of China; ^2^ Department of Urology, Shandong Provincial Hospital Affiliated to Shandong University (East Branch), Jinan, People's Republic of China; ^3^ Department of Urology, Lanling People's Hospital, Lanling, People's Republic of China; ^4^ Department of Urology, Dongying People's Hospital, Dongying, People's Republic of China; ^5^ Department of Urology, Yucheng People's Hospital, Yucheng, People's Republic of China; ^6^ Department of Urology, Guangrao County Hospital of traditional Chinese Medicine, Guangrao, People's Republic of China; ^7^ Department of Urology, Weihai Municipal Hospital, Weihai, People's Republic of China

**Keywords:** prostate cancer, risk assessment model, PSA, biopsy, North China

## Abstract

**Background:**

Prostate-specific antigen (PSA) is widely used for prostate cancer screening, but low specificity results in high false positive rates of prostate biopsies.

**Objective:**

To develop new risk assessment models to overcome the diagnostic limitation of PSA and reduce unnecessary prostate biopsies in North Chinese patients with 4–50 ng/mL PSA.

**Methods:**

A total of 702 patients in seven hospitals with 4–10 and 10–50 ng/mL PSA, respectively, who had undergone transrectal ultrasound-guided prostate biopsies, were assessed. Analysis-modeling stage for several clinical indexes related to prostate cancer and renal function was carried out. Multiple logistic regression analyses were used to develop new risk assessment models of prostate cancer for both PSA level ranges 4-10 and 10-50 ng/mL. External validation stage of the new models was performed to assess the necessity of biopsy.

**Results:**

The new models for both PSA ranges performed significantly better than PSA for detecting prostate cancers. Both models showed higher areas under the curves (0.937 and 0.873, respectively) compared with PSA alone (0.624 and 0.595), at pre-determined cut-off values of 0.1067 and 0.6183, respectively. Patients above the cut-off values were recommended for immediate biopsy, while the others were actively observed. External validation of the models showed significantly increased detection rates for prostate cancer (4-10 ng/mL group, 39.29% vs 17.79%, p=0.006; 10-50 ng/mL group, 71.83% vs 50.0%, p=0.015).

**Conclusions:**

We developed risk assessment models for North Chinese patients with 4–50 ng/mL PSA to reduce unnecessary prostate biopsies and increase the detection rate of prostate cancer.

## INTRODUCTION

Prostate cancer (PCa) is the most common cause of cancers in the Western population [1]. Although its incidence is lower in Asia, the relatively higher mortality rates and asymptomatic clinical features make early detection to remain a critical public health goal [1–5]. Transrectal ultrasound (TRUS)-guided prostate biopsy is currently the ‘gold standard’ for PCa diagnosis [6]. Nevertheless, there is a growing concern regarding the increasing incidence of serious infections, hematuria, hematospermia and bloody stool after biopsy [7].

Conventionally, the level of prostate-specific antigen (PSA), a serine protease secreted by prostate epithelial cells, determines whether a prostate biopsy should be performed [8]. However, the low sensitivity and specificity of PSA give rise to unnecessary biopsies, especially for patients with PSA ranging from 4 to 10 ng/mL, the so called “gray zone” [9–11]. In 2012, Wang et al. found TRUS-guided prostate biopsy positive diagnosis rates of 12.1%, 31.1%, 48.0% and 91.2% for PSA levels of <10, 10–20, 20–50 and >50 ng/mL group, respectively, in a cohort of Chinese patients [12]. Due to financial reasons, North Chinese individuals do not have so strong health examination consciousness as western people. They generally do not visit a doctor until they show lower urinary tract symptoms, which may account for the higher PSA level but lower positive diagnostic rates. Thus, the gray zone can be expanded to 4–50 ng/mL in North Chinese patients.

This study assessed clinical indexes in North Chinese patients with PSA levels of 4–10 and 10–50 ng/mL, respectively, who underwent biopsies. Then new risk assessment models of prostate cancer (RAM-PCa) were developed. These new models helped further formulate a reasonable follow-up strategy to overcome the limitations of PSA and the lack of health screening awareness. These findings might help increase PCa detection rates and reduce unnecessary prostate biopsies in North Chinese patients.

## RESULTS

This retrospective study evaluated 702 patients, divided into two groups based on PSA levels: 326 and 376 with 4–10 and 10–50 ng/mL PSA, respectively. Patient characteristics are shown in Tables 1 and 2, respectively. In patients with 4–10 ng/mL PSA, significant differences were found in age (P<0.001), digital rectal examination (DRE) (P<0.001), PSA (P=0.003), fPSA (P=0.016), f/tPSA (P<0.001), PSA density (PSAD, the ratio of PSA to PV) (P<0.001), creatinine (P=0.005), prostate volume (PV) (P=0.018), hypoechoic lesions in transabdominal ultrasound (HL-TAUS) (P<0.001), hypointense lesions in magnetic resonance imaging (HL-MRI) (<0.001) and breaking through the envelope of prostate in magnetic resonance imaging (BTEP-MRI) (P<0.001); no statistically significant difference was found in blood urea nitrogen (BUN). In patients with 10–50 ng/mL PSA, significant differences were obtained in age (P<0.001), DRE (P<0.001), PSA (P<0.001), PSAD (P<0.001), HL-TAUS (P<0.001), PV (P<0.001), HL-MRI (P<0.001) and BTEP-MRI (P<0.001), except fPSA, f/tPSA, creatinine and BUN. Older patients had an overtly higher incidence of prostate cancer. No statistically significant differences in fPSA and f/tPSA were found between the PCa and Non-PCa groups at the PSA 10-50ng/mL level, suggesting a lower diagnostic value with increasing PSA level. Surprisingly, patients with 4–10 ng/mL PSA in the PCa group showed lower creatinine levels than those in the Non-PCa group.

**Table 1:** Characteristics of patients with 4-10 ng/mL PSA in the analysis-modeling stage

Variables	Group		*P*
	Non-PCa	PCa	
No. of subjects, n(%)	268(82.21)	58(17.79)	
Age(year)	68.08±8.34	76.88±9.59	<0.001^a^
DRE, n(%)	42(15.67)	26(44.83)	<0.001^b^
PSA (ng/mL)	6.31±2.57	7.69±2.55	0.003^c^
fPSA(ng/mL)	1.13±0.79	0.88±0.96	0.016^c^
f/t PSA	0.18±0.10	0.14±0.12	<0.001^c^
PSAD	0.11±0.08	0.14±0.09	<0.001^c^
Creatinine (μmol/L)	83.25±20.06	77.00±22.00	0.005^c^
BUN(mmol/L)	5.80±2.00	6.20±2.40	0.217^c^
HL-TAUS, n(%)	18(6.72)	14(24.14)	<0.001^b^
PV(cm^3^)	57.67±38.42	53.28±35.04	0.018^c^
HL-MRI, n(%)	62(23.13)	30(51.72)	<0.001^b^
BTEP-MRI, n(%)	6(2.24)	12(20.69)	<0.001^b^

a: Student's t-test, prensented as Mean ± Standard Deviation (SD).

b: Chi-square test or Fisher exact probability test.

c: Rank sum test, prensented as Median ± Interquartile Range (IQR).

**Table 2:** Characteristics of patients with 10-50 ng/mL PSA in the analysis-modeling stage

Variables	Group		*P*
	Non-PCa	PCa	
No. of subjects, n(%)	188(50.00)	188(50.00)	
Age(year)	68.98±7.80	76.12±9.85	<0.001^a^
DRE, n(%)	46(24.47)	104(55.32)	<0.001^b^
PSA (ng/mL)	17.50±10.90	21.91±18.30	0.001^c^
fPSA(ng/mL)	2.47±1.60	2.69±2.68	0.098^c^
f/t PSA	0.15±0.08	0.14±0.11	0.374^c^
PSAD	0.25±0.24	0.46±0.57	<0.001^c^
Creatinine (μmol/L)	81.36±19.90	77.80±24.70	0.194^c^
BUN(mmol/L)	5.65±2.00	5.90±2.30	0.138^c^
HL-TAUS, n(%)	22(11.70)	70(37.23)	<0.001^b^
PV (mL)	71.24±42.58	45.82±34.27	<0.001^c^
HL-MRI, n(%)	64(34.04)	134(71.28)	<0.001^b^
BTEP-MRI, n(%)	6(3.19)	34(18.09)	<0.001^b^

a: Student's t-test, prensented as Mean ± SD.

b: Chi-square test or Fisher exact probability test.

c: Rank sum test, prensented as Median ± IQR.

In the PSA 4–10 ng/mL group, a multivariate logistic analysis with a backward elimination selection procedure was performed, evaluating age, ten-fold f/tPSA, ten-fold PSAD, creatinine, HL-MRI and BTEP-MRI (Tables 3 and 4). Meanwhile, in the PSA 10–50 ng/mL group, age, DRE, PSA, PV, HL-TAUS and HL-MRI were entered in multivariate logistic analysis (Tables 5 and 6). Thus, new models were established based on logistic analysis results. The equations for the risk assessment model of prostate cancer risk (RAM-PCaR) were defined as follows:

$$\begin{array}{l} RAM-PCaR_{\text{PSA }4-10}=\frac{e^{-12.165+0.191\times \text{Age}-1.489\times \text{Ten}-\text{foldf}/t\text{PSA}+0.838\times \text{Ten}-\text{fold PSAD}-0.033\times \text{creatinine}+1.073\times \text{HL}-\text{MRI}+3.375\times \text{BTEP}-\text{MRI}}}{1+e^{-12.165+0.191\times \text{Age}-1.489\times \text{Ten}-\text{foldf}/t\text{PSA}+0.838\times \text{Ten}-\text{fold PSAD}-0.033\times \text{creatinine}+1.073\times \text{HL}-\text{MRI}+3.375\times \text{BTEP}-\text{MRI}}}\\ RAM-PCaR_{\text{PSA }10-50}=\frac{e^{-9.728+0.111\times \text{Age}+1.439\times \text{DRE}+0.045\times \text{PSA}+1.258\times \text{HL}-\text{TAUS}-0.014\times \text{PV}+1.356\times \text{HL}-\text{MRI}}}{1+e^{-9.728+0.111\times \text{Age}+1.439\times \text{DRE}+0.045\times \text{PSA}+1.258\times \text{HL}-\text{TAUS}-0.014\times \text{PV}+1.356\times \text{HL}-\text{MRI}}} \end{array}$$

**Table 3:** Univariate logistic regression analysis of the PSA 4-10 ng/mL group

Variables	*OR*	95%*CI*		*P*
		Lower	Upper	
Age	1.133	1.088	1.180	<0.001*
DRE	4.372	2.368	8.074	<0.001*
PSA	1.272	1.081	1.498	0.004*
fPSA	0.597	0.338	1.054	0.075
Ten-fold f/t PSA	0.376	0.239	0.591	<0.001*
Ten-fold PSAD	2.341	1.606	3.411	<0.001*
Creatinine	0.981	0.965	0.997	0.024*
BUN	0.976	0.862	1.105	0.699
HL-TAUS	4.419	2.049	9.529	<0.001*
PV	0.989	0.978	1.000	0.047*
HL-MRI	3.560	1.977	6.410	<0.001*
BTEP-MRI	11.391	4.071	31.869	<0.001*

* *P*<0.05.

**Table 4:** Multivariate logistic regression analysis of the PSA 4-10 ng/mL group

Variables	*β*	*OR*	95%*CI*		*P*
			Lower	Upper	
Intercept	-12.165				<0.001
Age	0.191	1.211	1.146	1.279	<0.001
Ten-fold f/t PSA	-1.489	0.226	0.113	0.451	<0.001
Ten-fold PSAD	0.838	2.312	1.212	4.411	0.011
Creatinine	-0.033	0.967	0.945	0.990	0.006
HL-MRI	1.073	2.923	1.254	6.812	0.013
BTEP-MRI	3.375	29.233	6.782	126.016	<0.001

Six indexes above remained in the logistic analysis with a backward elimination selection procedure, and constructed an equation for RAM-PCa.

**Table 5:** Univariate logistic regression analysis of the PSA 10-50 ng/mL group

Variables	*OR*	95%*CI*		*P*
		Lower	Upper	
Age	1.096	1.068	1.126	<0.001*
DRE	3.822	2.463	5.932	<0.001*
PSA	1.043	1.024	1.064	<0.001*
fPSA	1.096	1.022	1.175	0.011*
Ten-fold f/t PSA	1.162	0.943	1.433	0.159
Ten-fold PSAD	1.328	1.214	1.453	<0.001*
Creatinine	0.996	0.990	1.002	0.224
BUN	1.036	0.950	1.130	0.425
HL-TAUS	4.476	2.624	7.635	<0.001*
PV	0.987	0.981	0.993	<0.001*
HL-MRI	4.808	3.106	7.441	<0.001*
BTEP-MRI	6.694	2.738	16.366	<0.001*

* *P*<0.05.

**Table 6:** Multivariate logistic regression analysis of the PSA 10-50 ng/mL group

Variables	*β*	*OR*	95%*CI*		*P*
			Lower	Upper	
Intercept	-9.728				<0.001
Age	0.111	1.118	1.082	1.155	<0.001
DRE	1.439	4.215	2.397	7.411	<0.001
PSA	0.045	1.046	1.018	1.074	0.001
HL-TURS	1.258	3.517	1.798	6.879	<0.001
PV	-0.014	0.986	0.979	0.993	<0.001
HL-MRI	1.356	3.882	2.231	6.755	<0.001

Six indexes above remained in the logistic analysis with a backward elimination selection procedure, and constructed an equation for RAM-PCa.

**Figure 1:**
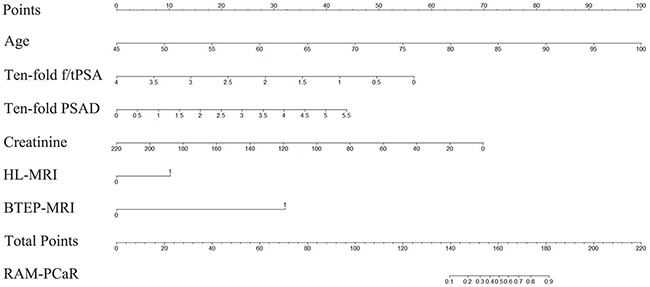
Nomogram of the RAM-PCa for the PSA 4-10 ng/mL group for predicting a positive prostate biopsy Individual values were placed on each variable axis to obtain the corresponding point on the ‘Point’ axis. The sum of these points was projected to the ‘Total Points’ axis to determine the probability of prostate cancer on the ‘RAM-PCaR’ axis.

**Figure 2:**
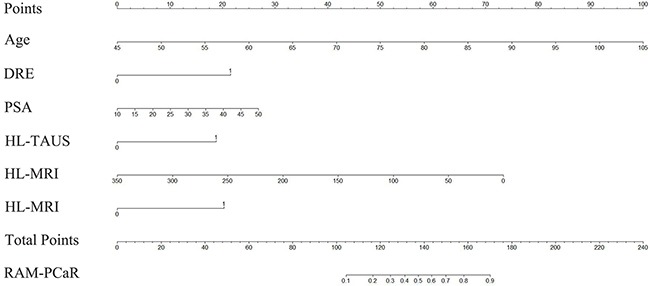
Nomogram of the RAM-PCa for the PSA 10-50 ng/mL group for predicting a positive prostate biopsy Individual values were placed on each variable axis, to obtain the corresponding point on the ‘Point’ axis. The sum of these points was projected to the ‘Total Points’ axis to determine the probability of prostate cancer on the ‘RAM-PCaR’ axis.

**Figure 3:**
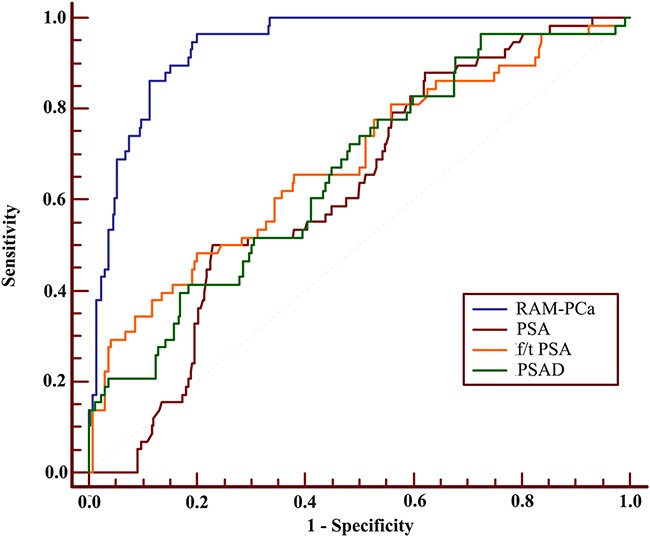
Receiver operating characteristic (ROC) curves of RAM-PCa, PSA, f/tPSA and PSAD for the PSA 4-10 ng/mL group AUC values of these indexes were 0.937, 0.624, 0.679 and 0.661, respectively. Sensitivities were 96.55%, 50.00%, 48.28% and 96.55%, respectively, for specificities of 80.00%, 77.15%, 79.85% and 28.09%, respectively.

**Figure 4:**
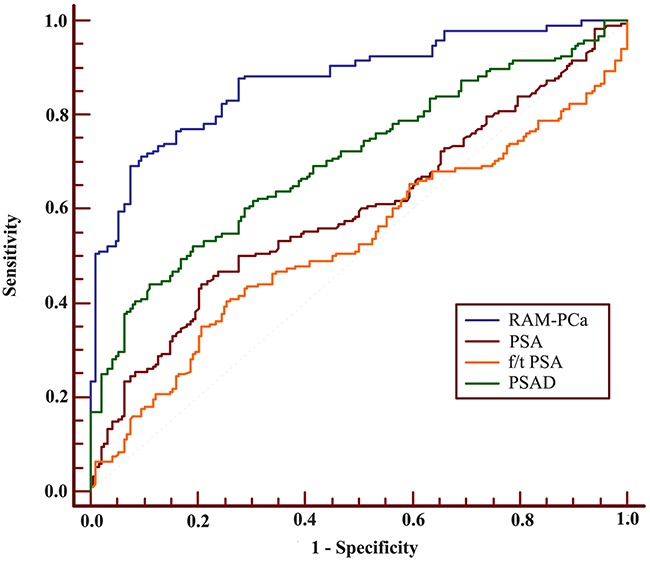
ROC of RAM-PCa, PSA, f/tPSA and PSAD for the PSA 10-50 ng/mL group AUC values of these indexes were 0.873, 0.595, 0.527 and 0.703, respectively. Sensitivities were 71.28%, 44.15%, 40.43% and 44.15%, respectively, with specificities of 90.43%, 79.26%, 74.47% and 88.83%, respectively.

**Figure 5:**
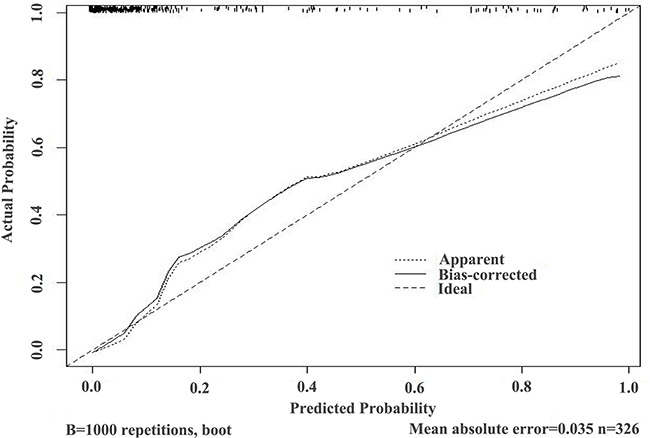
Validation curve of the predictive accuracy (93.7%) of RAM-PCa in the PSA 4-10 ng/mL group

**Figure 6:**
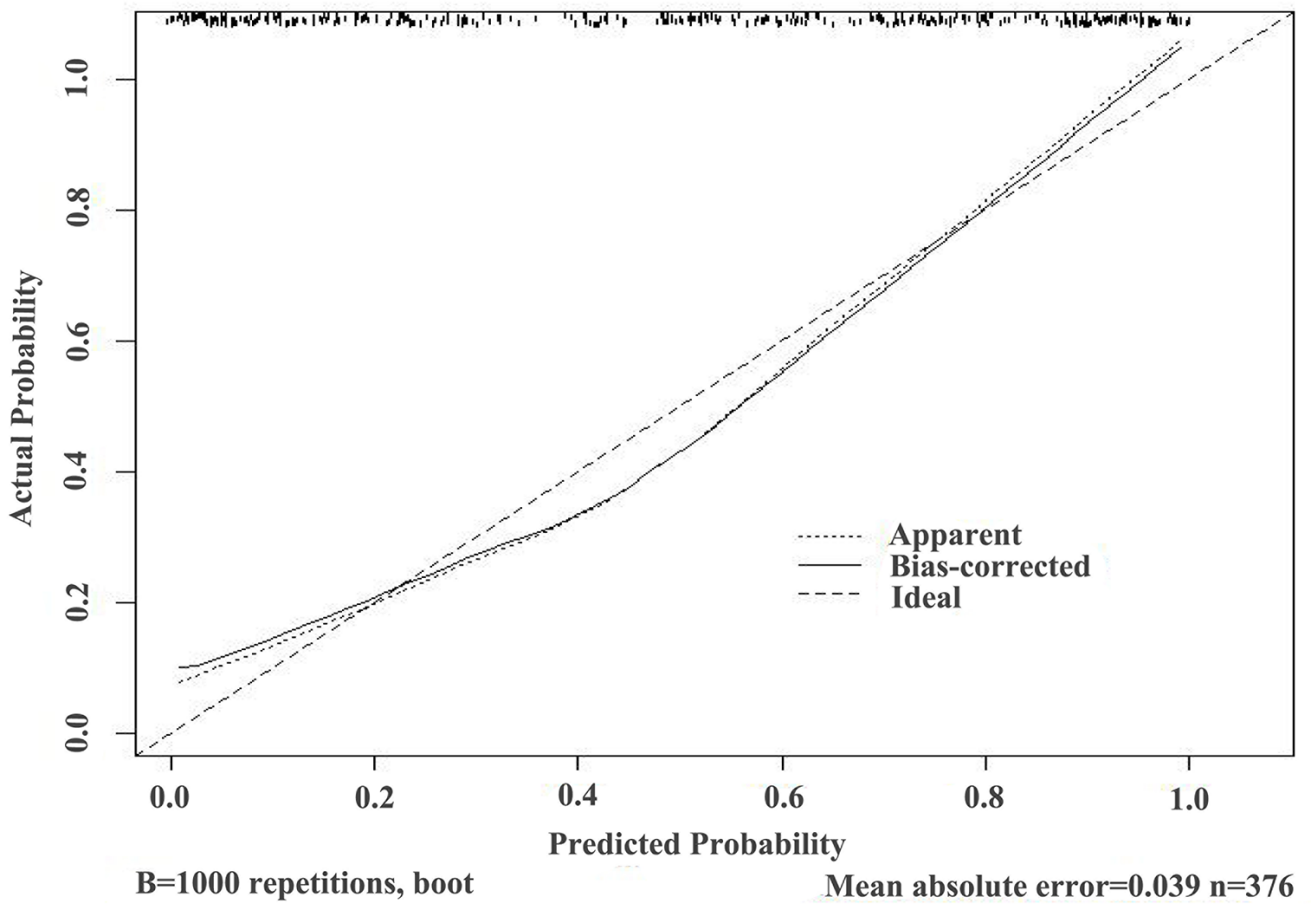
Validation curve of the predictive accuracy (87.3%) of RAM-PCa in the PSA 10-50 ng/mL group

These results provided sufficient information to support the generation of nomograms (Figures 1 and 2). Predictive efficiency and accuracy were quantified by determining the areas under the receiver operating characteristic curves (AUCs) (Figures 3 and 4). In the PSA 4–10 ng/mL group, the RAM-PCa yielded a higher AUC (0.937) compared with values obtained for PSA (0.624), f/tPSA (0.679) and PSAD (0.661) (Figure 3). In the new model, a cut-off value of 0.1067 was derived, yielding sensitivity and specificity of 96.55% and 80.00%, respectively, both higher than those of PSA, f/tPSA and PSAD. In the PSA 10–50 ng/mL group, the RAM-PCa had remarkably higher AUC (0.873), compared with values obtained for PSA (0.595), f/tPSA (0.527) and PSAD (0.703) (Figure 4). In this new model, a cut-off value of 0.6183 was determined, with sensitivity and specificity of 71.28% and 90.43%, respectively, both higher than those of PSA, f/tPSA and PSAD. The validation curves of these new models are shown in Figures 5 and 6. Using bootstrapping, the predictive accuracy values calculated by the Hosmer's concordance index were estimated at 0.937 and 0.873, respectively.

In the external validation study, 271 patients with PSA at 4–10 ng/mL and 10–50 ng/mL, respectively, in seven hospitals from January 2015 to July 2016, were assessed using the newly developed models. The high-risk group underwent biopsies while the remaining patients were under active monitoring. Statistical characteristics of the two stages are shown in Tables 7 and 8. For validation patients with 4–10 ng/mL PSA, the positive rate was improved from 17.79% to 39.29% (P=0.006) compared with the retrospective study. For validation patients with 10–50 ng/mL PSA, the positive rate was improved from 50.0% to 71.43% (P=0.015) compared with the retrospective stage. No significant differences were found in predictive indexes.

**Table 7:** Comparison of patient characteristics in two stages for the PSA 4-10 ng/mL group

Variables	Stage 1*	Stage 2*	*P*
No. of subjects, n(%)	326(75.64)	105(24.36)	
Age(year)	69.65±9.20	68.24±6.86	0.095^a^
DRE, n(%)	68(20.86)	16(15.24)	0.206^b^
PSA (ng/mL)	6.40±2.80	6.97±2.81	0.166^c^
fPSA(ng/mL)	1.11±0.81	1.14±0.81	0.464^c^
f/t PSA	0.18±0.07	0.19±0.08	0.333^a^
PSAD	0.12±0.08	0.11±0.08	0.264^c^
Creatinine (μmol/L)	82.00±21.68	78.00±18.00	0.423^c^
BUN(mmol/L)	5.80±2.00	5.60±1.80	0.430^c^
HL-TAUS, n(%)	32(9.82)	13(12.38)	0.455^b^
PV (mL)	56.78±36.80	59.62±43.68	0.070^c^
HL-MRI, n(%)	92(28.22)	24(22.86)	0.281^b^
BTEP-MRI, n(%)	18(5.52)	2(1.90)	0.126^b^
Biopsy case, n(%)	326(100.00)	28(26.67)	<0.001^b^
Positive cases, n(%)	58(17.79)	11(39.29)^#^	0.006

a: Student's t-test, prensented as Mean ± SD.

b: Chi-square test or Fisher exact probability test.

c: Rank sum test, prensented as Median ± IQR.

*: Stage 1: analysis-modeling stage; Stage 2: external validation stage.

^#^: The positive rate was discussed in high-risk patients in stage 2.

**Table 8:** Comparison of patient characteristics in two stages for the PSA 10-50 ng/mL group

Variables	Stage 1*	Stage 2*	*P*
No. of subjects, n(%)	376(69.37)	166(30.63)	
Age (year)	72.55±9.56	71.15±8.05	<0.079^a^
DRE, n(%)	150(39.89)	57(34.34)	0.220^b^
PSA (ng/mL)	18.75±14.66	17.69±12.50	0.459^c^
fPSA (ng/mL)	2.59±2.11	2.45±1.49	0.196^c^
f/t PSA	0.14±0.10	0.14±0.09	0.476^c^
PSAD	0.31±0.38	0.33±0.32	0.612^c^
Creatinine (μmol/L)	79.21±20.80	78.00±12.00	0.155^c^
BUN (mmol/L)	5.80±2.25	5.90±2.10	0.102^c^
HL-TAUS, n(%)	92(24.47)	31(18.67)	0.138^b^
PV (mL)	60.97±43.56	58.36±39.89	0.878^c^
HL-MRI, n(%)	198(52.66)	95(57.23)	0.325^b^
BTEP-MRI, n(%)	40(10.64)	12(7.23)	0.214^b^
Biopsy case, n(%)	376(100.00)	35(21.08)	<0.001^b^
Positive cases, n(%)	188(50.00)	25(71.43)^#^	0.015^b^

a: Student's t-test, prensented as Mean ± SD.

b: Chi-square test or Fisher exact probability test.

c: Rank sum test, prensented as Median ± IQR.

*: Stage 1: analysis-modeling stage; Stage 2: external validation stage.

^#^: The positive rate was discussed in high-risk patients in stage 2.

## DISCUSSION

Beside PCa, there are other physiological conditions, including benign prostatic hyperplasia, inflammation, infection, and trauma that increase PSA levels [8–11]. This could lead to unnecessary biopsies and over-diagnosis as well as over-treatment of PCa, causing pain, infection, bleeding, urinary obstruction, emotional distress and a waste of medical resources [13, 14]. Measures have been taken to reduce excessive biopsies in Western countries, including the addition of new predictive indexes such as [-2]proPSA (p2PSA), prostate health index (PHI), and prostate cancer antigen 3 (PCA3), to PSA screening models [11, 15, 16]. However, screening these new indexes on a large scale remains challenging for China. On the other hand, well-known models, such as prostate cancer prevention trial (PCPT) and European randomized study of screening for prostate cancer (ERSPC), based on Western populations, are not suitable for Chinese males owing to the overestimation of PCa risk due to population heterogeneity [17–20].

Instead of regular PSA checkups, Chinese individuals are usually examined for PSA when they have lower urinary tract symptoms, i.e., some individuals with high PSA levels but no serious lower urinary tract symptoms might not participate in PSA screening programs. This may account for the low positive diagnostic rate in the Chinese population at the same PSA level. In this study, the positive diagnosis rate of PSA 4–10 ng/mL was 17.79% while that of PSA 10–50 ng/mL was 50.00%. Thus, the gray zone could be expanded significantly from PSA 4–10 ng/mL to PSA 4–50 ng/mL among North Chinese individuals.

In both PSA 4–10 and 10–50 ng/mL groups of the analysis-modeling stage, patients with RAM-PCaR higher than respective cut-off values were assigned to the high-risk group while remaining constituted the low-risk group. Positive biopsy rates of high-risk and low-risk groups showed notable differences (50.90% vs. 0.93%, P<0.001 for PSA 4–10 ng/mL group; 87.58% vs. 24.22%, P<0.001 for PSA 10–50 ng/mL group). Specifically, in the low risk group of patients with 4–10 ng/mL PSA, only 2 patients were diagnosed with PCa out of 216 who underwent biopsy. The low-risk group of patients with 10–50 ng/mL PSA showed similar results, with 54 patients diagnosed with PCa out of 223 who underwent biopsy.

At the external validation stage, we prospectively verified the follow-up strategy in 105 patients with 4–10 ng/mL PSA and 166 individuals with 10–50 ng/mL PSA. A total of 28 out of 105 (26.67%) and 35 out of 166 (21.08%) patients underwent prostate biopsies due to RAM-PCaR higher than cutoff values. Interestingly, positive rates of PCa were improved significantly (PSA 4–10 ng/mL group, 17.79% vs. 39.29%, P=0.006; PSA 10–50 ng/mL group, 50.00% vs. 71.43%, P=0.015). In other words, a certain number of patients assigned to low risk groups could avoid immediate prostate biopsies and continue to enjoy good quality of life, but receiving active surveillance.

For retrospective study, creatinine acted as a protective factor in PSA 4-10 ng/ml group. Indeed, compared with the PCa group, the Non-PCa group had larger average prostate volumes (57.67 vs 53.28, p=0.018, Table 1), which might reflect more serious lower urinary tract symptoms, causing renal function impairment at a certain level. Current conventional assays show a positive correlation between blood creatinine and kidney damage [21]. Moreover, it can be concluded that PSA plays a more significant diagnostic role in patients with 10–50 ng/mL PSA, while f/t PSA and PSAD show obvious differences between the PCa and Non-PCa groups for individuals with 4–10 ng/mL PSA. This characteristic was in line with the Guidelines on Prostate Cancer of European Association of Urology using f/t PSA and PSAD to evaluate whether a prostate biopsy should be performed in the ‘grayzone’, PSA 4–10 ng/mL. And our previous study suggested that PSA other than PSAD, remained valuable predictor of the pathologic stage of PCa [22]. Thus, further study could be done to investigate the new model using PSA as well as other clinical indexes to predict the pathologic stage of PCa.

This study included 973 patients from January 2010 to July 2016 from seven hospitals across North China, with a population accounting for 25% of the whole country. In addition to the large-scale and multiple institutional sampling, this study expanded the gray-zone from PSA 4–10 ng/mL to PSA 4–50 ng/mL for North Chinese individuals. Thus, a widespread application of the current findings can be made, significantly reducing the unnecessary biopsies for such regions, where the economic foundation, cultural basis, and health awareness are not strong. Moreover, creatinine showed obvious statistical differences between the PCa and Non-PCa groups in 4-10 ng/mL PSA model and was absorbed in risk assessment models at the first time.

The study had several limitations. First, the total number of patients, 702 patients for analysis-modeling stage and 271 for external validation stage, was relatively small. In addition, prostate volumes were measured by MRI, with potential errors in the calculated outcome of volume formulation. Thirdly, the RAM-PCa receiver operating characteristic curve (ROC) for the PSA 10-50 ng/mL group showed lower sensitivity compared with that of the PSA 4-10 ng/mL group (71.28% vs 96.55%), indicating that a certain number of individuals would be misdiagnosed. However, larger sample size, more definite group and additional correlated indexes are being assessed by our team in order to establish a more convincing model in the future.

## MATERIALS AND METHODS

### Participants

The present study included two stages: analysis-modeling stage and external validation stage. The former was a multi-institutional retrospective study to develop risk assessment models. It included 702 patients with elevated PSA levels from 4 to 50 ng/mL, who had undergone a TRUS-guided prostate biopsy in seven hospitals (listed below) across North China, from January 2010 to December 2014. In the external validation stage, 271 patients with PSA levels ranging from 4 to 50 ng/mL from the same institutions, from January 2015 to July 2016, were assessed to advise regarding biopsy performance using the new model. In both stages, patients with a history of urinary infection, urinary tract trauma, transurethral prostate resection or prostate biopsy were excluded. The seven hospitals included Shandong Provincial Hospital Affiliated to Shandong University, Shandong Provincial Hospital Affiliated to Shandong University (East Branch), Lanling People's Hospital, Dongying People's Hospital, Yucheng People's Hospital, Guangrao County Hospital of traditional Chinese Medicine and Weihai Municipal Hospital.

### Study design

#### Analysis-modeling stage

This stage included 702 patients, with 326 and 376 in the PSA 4–10 ng/mL and 10–50 ng/mL groups, respectively. The patiens underwent TRUS-guided conventional systemic 12-core biopsy, in addition to a special core, derived from hypoechoic lesions in ultrasound, hypointense lesions in magnetic resonance imaging or the prostatic apex. According to biopsy pathology, each group was divided into two subgroups, PCa and Non-PCa. Basic clinical indexes, including age, PSA, fPSA, f/tPSA, DRE, PV (obtained by MRI of the prostate, as PV = 1/6 ×π× transverse diameter × anteroposterior diameter ×cephalocaudal diameter), PSAD, two renal function indexes [BUN and creatinine], HL-TAUS, HL-MRI, and BTEP-MRI were collected. Then differences in the above indexes between the PCa and Non-PCa groups were assessed by Student's t-test, rank-sum test, chi-square test, or Fisher exact probability test. Multiple logistic regression analyses using a backward elimination selection procedure were applied to build risk assessment models followed by the appraisal of diagnostic efficiency (ROC) and accuracy (validation curve). Furthermore, the normograms assisted in predicting biopsy outcome, visually and intuitively.

#### External validation stage

In this stage, 271 patients were prospectively evaluated, including 105 and 166 with PSA levels of 4–10 ng/mL and 10–50 ng/mL, respectively, using the newly developed models. Based on the comparison between the cutoff values of ROC and the individual values calculated from the RAM-PCa equation, patients were classified into high-risk and low-risk groups, respectively. High-risk patients were advised to undergo prostate biopsies, and low-risk group was advised to undergo continuous observation. Finally, biopsy rates and other common indexes were assessed between the two stages, while the detection rate of PCa was compared between first stage groups and the high-risk group in the second stage.

### Statistical analysis

Statistical analyses were carried out with the SPSS 19.0 software, R software version 2.15.0, and MedCalc software version 11.4.2.0. Differences in patient characteristics were analyzed by Student's t-test for continuous variables with a Gaussian distribution, rank-sum test for continuous variables with non-normal distribution, and chi-square test or Fisher's exact probability test for categorical variables. After selecting potential predictive factors, multiple logistic regression analyses with a backward elimination selection procedure were used to develop the new risk assessment models. ROC curves were used to evaluate the efficiency of the models and those of other indexes such as PSA, f/tPSA, and PSAD. The Hosmer-Lemeshow test using the concordance index on 1000 bootstrapped samples was employed for the validation of the new models.

## CONCLUSIONS

Overall, we developed new models to assess whether TRUS-guided prostate biopsy should be performed in North Chinese patients with PSA level ranges 4 and 50 ng/mL. In addition, we developed a follow-up strategy for such regions lacking new PCa screening methods and health examination awareness. The current findings could help reduce the number of unnecessary prostate biopsies, also increasing the detection rate of PCa without delaying diagnosis and treatment for North Chinese patients.

## Abbreviations

PCa: prostate cancer
PSA: prostate-specific antigen
TRUS: Transrectal ultrasound
fPSA: free PSA
f/tPSA: free/total PSA
PSAD: PSA density
PV: prostate volume
HL-TAUS: hypoechoic lesions in transabdominal ultrasound
HL-MRI: hypointense lesions in magnetic resonance imaging
BTEP-MRI: breaking through the envelope of prostate in magnetic resonance imaging
RAM-PCa: risk assessment models of prostate cancer
RAM-PCaR: risk of risk assessment model for prostate cancer
ROC: receiver operating characteristic curve
AUC: areas under curve
p2PSA: [-2]proPSA
PHI: prostate health index
PCA3: prostate cancer antigen 3
PCPT: prostate cancer prevention trial
ERSPC: European randomized study of screening for prostate cancer
SD: Standard Deviation
IQR: Interquartile Range.
